# Necrotizing Soft Tissue Infections: Surgeon's Prospective

**DOI:** 10.1155/2013/609628

**Published:** 2013-12-24

**Authors:** Shashi Prakash Mishra, Shivanshu Singh, Sanjeev Kumar Gupta

**Affiliations:** Department of General Surgery, Institute of Medical Sciences, Banaras Hindu University, Varanasi 221005, India

## Abstract

Necrotizing soft tissue infections (NSTIs) are fulminant infections of any layer of the soft tissue compartment associated with widespread necrosis and systemic toxicity. Delay in diagnosing and treating these infections increases the risk of mortality. Early and aggressive surgical debridement with support for the failing organs significantly improves the survival. Although there are different forms of NSTIs like Fournier's gangrene or clostridial myonecrosis, the most important fact is that they share common pathophysiology and principles of treatment. The current paper summarizes the pathophysiology, clinical features, the diagnostic workup required and the treatment principles to manage these cases.

## 1. Introduction

Necrotizing soft tissue infections (NSTIs) are fulminant infections of any layer of the soft tissue compartment associated with widespread necrosis, systemic toxicity and a high mortality rate if not treated early. These infections were first described by Jones in 1871 and at that time they were termed “hospital gangrene” [1]. The term “necrotizing soft tissue infections” has been emphasized to encompass all necrotizing infections and advocate an approach to all of them that uses the same principles for diagnostic and treatment strategies [2].

## 2. The Current Scenario

The incidence of NSTIs in the United States of America is estimated to be around 500–1500 cases per year [3]. The mortality rates have been reported to be around 25%, although it is higher in some studies [4].

## 3. Classification of NSTIs

The Food and Drug Administration (FDA) has classified skin and soft tissue infections in to two categories, namely, the complicated infections [5, 6].

Based on the microbiological characteristics, NSTIs are broadly classified into three types.

*Type I Infections*. Type I infections are the most common forms accounting for 80% or more of all the NSTIs, for example, Fournier's gangrene and Ludwig's angina [7]. These are mostly polymicrobial infections having a combination of aerobes, anaerobes and facultative aerobes/anaerobes. The common aerobic species isolated from these infections are *streptococci, staphylococci, enterococci*, and the family of gram-negative rods. *Bacteroides* species are the most common anaerobes involved.

*Type II Infections*. Type II infections are usually mono-microbial and usually follow a minor injury accounting for 10–15% of all NSTIs [8, 9]. Common organisms involved are the Group A beta-hemolytic streptococcus or the *S. aureus.*

*Type III Infections*. These account for less than 5% of all NSTIs and clostridial gas forming myonecrosis is the prototype of type III infections, most commonly caused by *Cl. perfringens* [10–12].

Necrotizing soft tissue infections are also caused by *Vibrio vulnificus* and *Aeromonas hydrophila* and various fungi as *Mucor*, *Rhizopus,* or *Rhizomucor* [13–15].

## 4. The Pathogenesis

NSTI is the condition where the microbial virulence overweighs the host defense system. Impaired host immunity or local tissue hypoxia as in atherosclerosis, burns, cancer or other immunocompromised states, chronic alcoholism, corticosteroid use, diabetes mellitus, hypoalbuminemia, intravenous drug abuse, malnutrition, obesity, occult diverticulitis, peripheral vascular disease, postoperative infection, and trauma predispose to NSTI. Clostridial infections can occur following bowel surgery, heroine drug abuse or in obstetrical complications [16–18].

The streptococcal capsule and protein M, protein F, streptolysin O, hyaluronidase, streptokinase, and pyrogenic exotoxins have their specific roles to play in the pathogenesis of streptococcal infections [19–22]. The staphylococcal Panton-Valentine leukocidin, alpha-hemolysin, and phenol-soluble modulins are responsible for the pathogenesis of staphylococcal infections [23–25]. The *Clostridium* causes its deleterious effects by clostridial alpha, theta, and kappa toxins. The fungi proliferate more in the presence of chelated iron [26]. Patients on iron chelator therapy have increased risk of fungal infection.

The widespread tissue destruction and gangrene as a result of vascular thrombosis along with the systemic release of toxins and the adverse effects of massive cytokine release contribute to multiorgan failure and a high mortality rate.

## 5. The Clinical Features: When to Suspect an NSTI

Perhaps the biggest hurdle in early diagnosis and management of an NSTI is the diagnosis. The commonly involved sites are the extremities (36–55%), trunk (18–64%) and perineum (up to 36%) [27–31]. Events commonly predisposing patients to NSTIs include mild trauma, insect bites, drug reactions, illicit drug injections, perirectal abscesses, major traumas, and surgical procedures [28]. Although patients are usually having an underlying risk factor as discussed, 30% of the NSTIs do occur in healthy individuals [32].

The initial nonspecific signs such as tenderness, swelling, erythema, and pain at the affected site mimic nonsevere soft tissue infections such as cellulitis and erysipelas [27, 33, 34]. Among them, the cardinal manifestation is severe pain at onset out of proportion to physical findings [35–37]. *Vibrio* and *Aeromonas* are well known waterborne organisms that cause NSTI with high mortality through infection of patients with chronic illnesses, especially in the liver. Careful history taking concerning seawater exposure or fish stings [38] with liver or spleen dysfunction is the key to narrowing down the candidate organism.

Fever (>38°C) is found in around 44% of the cases and tachycardia (>100 beats/min) is usually found in 59% cases. Infected sites have erythema (80%), induration (66%), tenderness (54%), fluctuance (35%), skin necrosis (23%), and bullae (11%) [39]. The initial physical findings of NSTI are usually erythematous and ecchymotic skin lesions, but these may rapidly evolve into hemorrhagic bullae, which indicate the occlusion of deep blood vessels in the fascia or muscle compartments. Extensive subcutaneous gas effusions detected as crepitation on palpation characterize the clostridial infections. A seropurulent, foul smelling “dish-water” like discharge characterizes NSTIs. If not suspected and managed, the disease progresses in to sepsis, septic shock, and multiorgan dysfunction syndrome.

## 6. Establishing the Diagnosis of NSTI

The most important feature that needs to be identified in such soft tissue infections is the presence of necrotizing component. This identifies the patients requiring surgical debridement and supportive care. The first step towards diagnosis is to have a high index of suspicion [3]. Another problem is the rapid progression of the disease once the typical late clinical features get established, so both clinical clues and diagnostic tools should be used in combination to make an early diagnosis [40].

### 6.1. Laboratory Investigations

The usual laboratory findings at the time of admission are WBC count more than 14 × 10^9^/L, serum sodium less than 135 nmol/L, BUN more than 15 mg/dL, thrombocytopenia, hypocalcaemia hypoproteinemia, hypoalbuminemia, elevated serum glucose, and hemoglobin less than 10 g/dL, hypocholesterolemia and serum creatinine more than 2 mg/dL, and increased blood lactate. The last three findings are associated with increased mortality [41]. A laboratory risk indicator for necrotizing fasciitis score also known as LRINEC score was devised [42] by assigning scores to a certain set of laboratory variables (Table 1). The six independent variables included were serum CRP levels, total leukocyte count, hemoglobin level, serum sodium, serum creatinine, and glucose levels. The total score ranges from 0 to 13 (Table 2) and patients could be categorized into three groups based on the probability of NSTI. It was found that for intermediate and high-risk patients, the score had a positive predictive value of 92% and a negative predictive value of 96%. However, LRINEC score is useful only in the context of a diagnosed or strongly suspected severe soft tissue infection.

### 6.2. Imaging Tools

Imaging tools are useful if the diagnosis of NSTI is doubtful. These are though non invasive but sometimes they unnecessarily delay the diagnosis of clinically evident NSTI. Presence of subcutaneous gas is a typical X-ray finding but is not a sensitive finding [3].

The computed tomography scan findings suggestive of NSTI include the extent of abnormal soft tissue gas dissecting along the fascial planes, fascial stranding, and asymmetric thickening of fascial planes [43]. The sensitivity of CT to identify NSTI is 100%, specificity is 81%, positive predictive value is 76%, and negative predictive value is 100% [44].

The gadolinium contrast enhanced magnetic resonance imaging is useful in stable, conscious, cooperative, and not seriously septic patients [45]. The findings significantly suggestive of necrotizing infection include thick (≥3 mm) abnormal signal intensity on fat-suppressed T2-weighted images, low signal intensity in the deep fascia on fat-suppressed T1-weighted images, a focal or diffuse nonenhancing portion in the area of abnormal signal intensity in the deep fascia, extensive involvement of the deep fascia, and involvement of three or more compartments in one extremity.

### 6.3. Microscopic and Macroscopic Tools

Examination of a frozen section of a tissue biopsy specimen from the necrotic deep fascia and possibly the muscle has been recommended as means for early diagnosis, thereby reducing mortality in NSTI [46–48]. The characteristic findings include subcutaneous necrosis, polymorphonuclear cell infiltration, fibrinous vascular thrombosis with necrosis, microorganisms within the destroyed fascia, and dermis. Gram staining may also reveal gram-positive clostridial bacilli. Histopathology can also identify fungal infection and invasive fungal infection with vascular thrombosis. Fine-needle or large-bore needle aspiration is another method by which to establish the diagnosis and direct antimicrobial therapy [49].

The characteristic features of NSTI on exploration of compromised area include grey necrotic tissue, lack of bleeding, thrombosed vessels, “dishwater” pus, noncontracting muscle, and a positive “finger test” (lack of resistance to finger dissection in normally adherent tissues). Once NSTI is confirmed, the incision is extended and additional debridement is performed. It has been recommended to perform exploration of the involved area through an operation whenever there is doubt and likelihood for NSTI.

### 6.4. Microbiological Culture

NSTIs are commonly polymicrobial infections with a synergistic interaction between aerobes and anaerobes, so a good microbiological culture can guide therapy and improve the outcome.

## 7. Treatment

Care for patients with necrotizing soft tissue infection requires a team approach with expertise from critical care, surgery, reconstructive surgery, and rehabilitation specialists [50]. The four treatment principles are fluid resuscitation and correction of electrolyte and acid-base imbalance, early initiation of antibiotics, surgical debridement of the affected area, and supportive measures for organ failure [7]. In addition to antimicrobial therapy, complete debridement of infected tissue is key to successful treatment [3].

### 7.1. Fluid Resuscitation and Correction of Electrolyte and Acid-Base Abnormalities

There is always a state of fluid depletion due to infection, systemic inflammatory response, and raw area following debridement which may predispose to multi-organ failure, thereby increasing the mortality and morbidity.

The first line fluids are the crystalloids; however, laboratory value of serum electrolytes, renal function, and hepatic functions needs to be assessed before selecting the fluid of choice. Fresh frozen plasma and platelet transfusions are reserved for patients who have coagulopathy and thrombocytopenia, respectively.

The common electrolyte imbalances requiring correction are hyponatremia and hypocalcemia. Central venous pressure and urine output are measured to determine the adequacy of fluid resuscitation.

### 7.2. The Antimicrobial Therapy

Early initiation of antimicrobial therapy is essential and adjunctive to debridement. The current empirical antimicrobial regimes [23, 51] advocate use of Piperacillin-Tazobactam at 3.375 gm every six hours with clindamycin 400–600 mg every four to six hours with ciprofloxacin 400 mg every twelve hours in type I infections. For type II infections, penicillin (2–4 million units very four to six hours) with clindamycin is recommended. Injection linezolid (600 mg every 12 hours) or vancomycin (30 mg/kg/day in two divided doses) may be considered in those allergic to penicillin. For type III clostridial infections, combination of penicillin with clindamycin is effective. In case of *Vibrio* or *Aeromonas *infection, doxycycline in a dose of 1 gram every twelve hours is effective. Clindamycin suppresses the toxin production by *S. aureus, *hemolytic *streptococci*, and clostridia and should be included when these organisms are present or suspected and it is specially indicated for all patients having hypotension, coagulopathy, or organ system failure [52]. Certain drugs like vancomycin may require dose adjustments in case of renal or hepatic failure. The final course of antimicrobial therapy should be based on the culture sensitivity reports. In cases of fungal infections, intravenous amphotericin B is given.

### 7.3. Surgical Debridement

Researchers have clearly shown the impact of early and complete debridement on final outcome in patients with NSTI [29, 30, 53]. The area of necrosis often extends beyond what is anticipated based on external appearance of the skin due to thrombosis of the dermal capillary beds that precedes skin necrosis. All obviously necrotic skin, subcutaneous tissue, fascia, and muscle must be excised. When there is crepitance present over an area of normal appearing skin, an exploratory incision should be made through the involved area to determine whether the underlying tissues are viable. The presence of soft tissue gas does not mandate excision as long as the underlying tissues are viable. This crepitance often resolves after the necrotic tissue is removed.

Amputation is done in 25–50% of the cases where the affected extremity is either nonviable or would not be functional following debridement [54] Other surgical procedures required may include diversion colostomy to prevent perineal soiling in cases involving perineum and patient is having rectal incontinence [29]. Orchiectomy is rarely required in cases of Fournier's gangrene since the blood supply to the testicles is usually preserved [55].

### 7.4. Wound Care

Reexploration of the wound is done every 6–48 hours and redebridement may be required until all the necrotic tissue has been removed. The use of a vacuum- assisted closure system can accelerate wound contraction and time to closure, but this should not be initiated until the necrosis and infection are controlled and granulation tissue has appeared [56]. The exposed area should not be covered with skin grafts before the acute infection has resolved. Once the infection has resolved and a healthy granulation tissue appears, wound closure may be accomplished by skin grafts, flap cover, by direct approximation, or with the help of skin regenerative matrices.

### 7.5. Organ Support Measures

Patients who are hypotensive after appropriate fluid resuscitation should receive vasopressors and should be monitored in an intensive care unit [50]. Extracorporeal membrane oxygenation is required in patients with cardiopulmonary failure [57]. Often, the best way to support organ systems is by rapidly controlling the infectious process, which then allows the physiologic environment to return to normal.

### 7.6. Novel Therapeutic Strategies

Role of hyperbaric oxygen therapy and intravenous immunoglobulin in the management of NSTI has been an area of interest for many researchers. Hyperbaric oxygen (HBO) results in greater oxygen partial pressures in perfused body tissues [58]. It has been shown to inhibit exotoxin production by clostridia in experimental studies [59]. The results of human studies are conflicting and most prospective studies fail to show a benefit for HBO in these circumstances [7]. Intravenous immunoglobulin (IVIG) has been advocated for the treatment of NSTI due to *streptococci* and *staphylococci* [60]. The evidence supporting the use of IVIG for treatment of NSTI is controversial, although there are retrospective as well as prospective studies that have demonstrated some benefit. Use of drotecogin alpha has been shown to improve outcome in selected patients with severe sepsis. Some studies have shown it's survival advantage in cases of NSTI [61].

### 7.7. Build-Up following Initial Therapies

Wound healing and immune activity along with sepsis create a catabolic state in the body. Therefore, nutrition plays a vital role in the recovery of these cases. Vitamins A, C, and D and minerals such as zinc should be provided since these can promote wound healing.

## 8. Prognosis

In the past, the mortality rate in NSTI was high as high as 46% [1]. A decade ago, pooled analysis determined it to be nearly 34% [29]. Currently, the reported mortality rate in National Surgical Quality Improvement Program is only 12% [62]. A delay of surgical treatment of more than 12 hours is associated with an increased number of surgical debridement and higher incidence of septic shock and acute renal failure [63].

Anaya and colleagues [51] created a score to categorize patients according to the risk of mortality (Table 3). Variables included in the score were age >50 years; WBC count >40,000 cells/mm^3^; hematocrit >50%; heart rate >110 beats/min; temperature <36°C; and creatinine level >1.5 mg/dL. Patients could be categorized in 3 groups, according to the risk of mortality.

## 9. Conclusion

Necrotizing soft tissue infections are fulminant polymicrobial soft tissue infections with a high mortality rate. Understanding the pathophysiology, the principles of treatment and the role of early and repeated surgical debridement will improve the outcome of these cases. Novel therapeutic measures have limited applicability with questionable benefit. The timely initiation of fluid and electrolyte management, antimicrobial therapy, and surgical debridement with wound care and support for organ failure has markedly reduced the mortality in necrotizing soft tissue infections.

## Figures and Tables

**Table 1:** Laboratory risk indicator for necrotizing fasciitis score—LRINEC [55].

Laboratory parameter and its value	Assigned score
C- reactive protein (mg/lt)	
<150	0
≥150	4
TLC (cells/cu mm)	
<15000	0
15000–25000	1
>25000	2
Hemoglobin (gm/dL)	
>13.5	0
11–13.5	1
<11	2
Serum sodium	
135 or more	0
<135	2
Serum creatinine (mg/dL)	
≤1.6	0
>1.6	2
Glucose (mg/dL)	
≤180	0
>180	1

TLC: total leukocyte count.

**Table 2:** Patient categories within LRINEC score according to the likelihood of necrotizing soft tissue infection (NSTI) [55].

Risk category	LRINEC score	Possibility of NSTI %
Low	≤5	<50%
Intermediate	6-7	50–75%
High	≥8	>75%

**Table 3:** Clinical score predictive of death for patients with necrotizing soft tissue infections [51].

Parameter		Score
Heart rate more than 110 beats per minute		1
Temperature less than 36°C		1
Serum creatinine more than 1.5 mg/dL		1
Age more than 50 years		3
White blood cell count more than 40,000/mcL		3
Hematocrit more than 50%		3

Group category	Number of points	Percentage mortality

1	0–2	6%
2	3–5	24%
3	6 or more	88%
